# A unique stenosis in saphenous vein graft visualized by optical coherence tomography

**DOI:** 10.1007/s00380-013-0362-x

**Published:** 2013-05-28

**Authors:** Yorihiko Koeda, Tomonori Itoh, Tetsuya Fusazaki, Motoyuki Nakamura, Yoshihiro Morino

**Affiliations:** 1 Division of Cardiology, Department of Internal Medicine and Memorial Heart Center, Iwate Medical University School of Medicine, 1-2-1 Chuo-dori, Morioka, Iwate 020-8505 Japan; 2 Division of Cardiovascular Medicine, Nephrology and Endocrinology, Department of Internal Medicine, School of Medicine, Iwate Medical University, 1-2-1 Chuo-dori, Morioka, Iwate 020-8505 Japan

**Keywords:** Optical coherence tomography, Saphenous vein graft, Venous valve, Coronary artery bypass graft

## Abstract

We present a case of a unique stenosis in a 12-year-old saphenous vein graft (SVG), to the right coronary artery, which was visualized by optical coherence tomography (OCT), before percutaneous coronary intervention. The patient was an 80-year-old man in whom the stenosis was documented by area-detector coronary computed tomography. OCT imaging demonstrated that the culprit lesion was a venous valve containing a thrombus before preintervention imaging. Coronary stenting was performed with a distal protection device, and pathologic examination of the aspirate verified the OCT findings. Coronary angiography 12 years previously, just after coronary artery bypass surgery (CABG), had shown a completely normal SVG without any suspicion of a venous valve. These OCT images suggested the possibility that the culprit lesion was an “upside down” venous valve that was not visualized by angiography just after surgery, but could be a cause of late SVG stenosis following CABG. OCT imaging is very useful for clarifying the etiology of the stenosis in cases of ambiguous angiographic lesions.

Coronary artery bypass grafting (CABG) is established as a means of restoring blood flow to areas of myocardial ischemia in patients with coronary artery disease. Owing to substantial advances in therapeutic strategies and optimal medical therapy, clinical outcomes after CABG have been improved even in octogenarians [1]. However, in patients who require coronary angiography after CABG, coronary risk factors tend to be poorly controlled, and these patients are at high risk for adverse cardiac events [2]. Therefore, extensive assessment and management would be essential to improve outcomes in patients with prior CABG requiring coronary angiograms. Although saphenous vein grafting (SVG) is generally used, it has lower patency rates compared with internal thoracic artery implantation, which is referred to as vein graft disease. Various factors are pointed out as a cause of vein graft disease [3]. In this case report we describe the occurrence of a unique SVG stenosis detected by OCT imaging, and suggest that structural alterations in relation to venous valves may be responsible for other cases of vein graft disease as well.

An 80-year-old Japanese man with hypertension and dyslipidemia, with a history of acute inferior myocardial infarction 12 years previously, had undergone CABG to treat triple-vessel disease. His grafts were as follows: aorta → SVG → right coronary artery (RCA), right internal thoracic artery graft → left anterior descending artery, left internal thoracic artery graft → left circumflex coronary artery. Three months following CABG he again underwent coronary angiography (CAG) as a routine follow up (there were no clinical symptoms), which did not disclose any stenosis of the SVG, or of any other graft (Fig. 1). Twelve years later, the patient underwent computed tomographic coronary angiography (CTA) in ambulatory care, because he experienced chest oppression during effort. A stenosis was documented at the mid portion of the SVG (Fig. 2). CAG revealed a nonsignificant stenosis at the mid portion of the SVG to RCA (Fig. 3A). We decided to use intravascular ultrasonography (IVUS) (Fig. 3B, C) and optical coherence tomography (OCT) imaging (Fig. 3D, E) to clarify the nature of the SVG stenosis. The long-axis OCT image (Fig. 3E) disclosed morphology possibly representing a venous valve at the site of the culprit lesion, leading us to speculate that the graft might have been attached upside down during CABG 12 years previously. According to the operative note at the time, surgeons had not intentionally selected a nonreversed SVG with some modification [4, 5] to the venous valve. There was no indication of the direction of the SVG in the record, or that it had been modified. This in turn may have caused thrombus formation and graft stenosis at the site of a venous valve. Compared with venous valves of normal SVGs [6], the valve in this case was clearly thickened. Venous valves differ in size or thickness depending on several factors, such as position, vessel diameter, and dynamic stress caused by blood. Thickening of the venous valve in an upside-down SVG might be due to its position, which is against the direction of the blood flow, leading to thrombus formation. This unique OCT image should be differentiated from spontaneous dissection or a ruptured atheromatous plaque. However, after checking multiple long-axis OCT angles, we believe that it corresponds to a venous valve because of its short symmetric and regular structure. Nevertheless, it is not clear from the three-dimensional OCT images whether the venous valve was indeed upside-down (Fig. 4). On the other hand, IVUS was not helpful in clarifying the nature of this lesion. Percutaneous coronary intervention (PCI) was performed using a distal protection device, Filtrap (Osaka, Japan), and a drug-eluting stent, Nobori (3.5 × 16 mm) (Terumo, Tokyo, Japan) (Fig. 3F). Thrombus was retained by the distal protection device (Fig. 5), and consisted of red blood cells and platelets. There were also a few neutrophils, eosinophils, phosphotungstic acid hematoxylin (PTAH)-positive fibrin, and CD68-positive macrophages. These pathologic findings are consistent with organized chronic thrombus. The presence of the venous valve may have played a role in the formation of organized thrombus, which in turn may have affected the thickened appearance of the valve. It is known that SVGs deteriorate following CABG (vein graft disease), with half being totally occluded or severely stenosed within 10 years [7, 8]. However, this patient’s vein graft was patent for 12 years, a relatively favorable outcome.

**Fig. 1:**
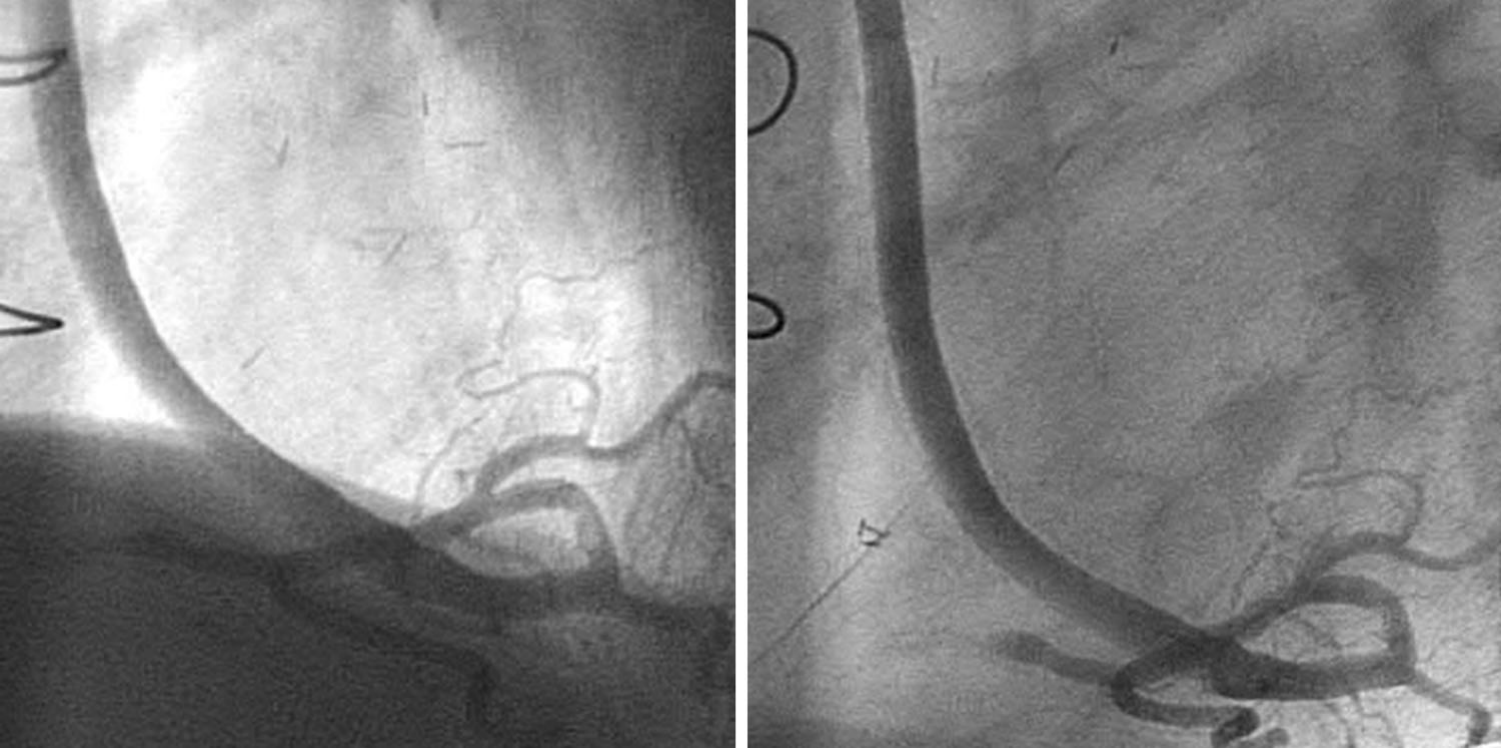
Saphenous vein grafting images of the graft angiography 12 years previously. *Left* anterior-posterior view. *Right* left anterior oblique view

**Fig. 2:**
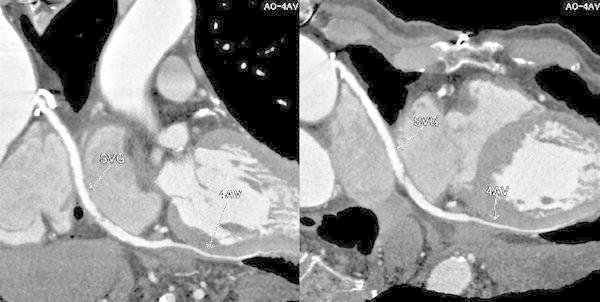
Saphenous vein grafting images of the computed tomographic angiography. A stenosis at the mid portion of the saphenous vein graft

**Fig. 3:**
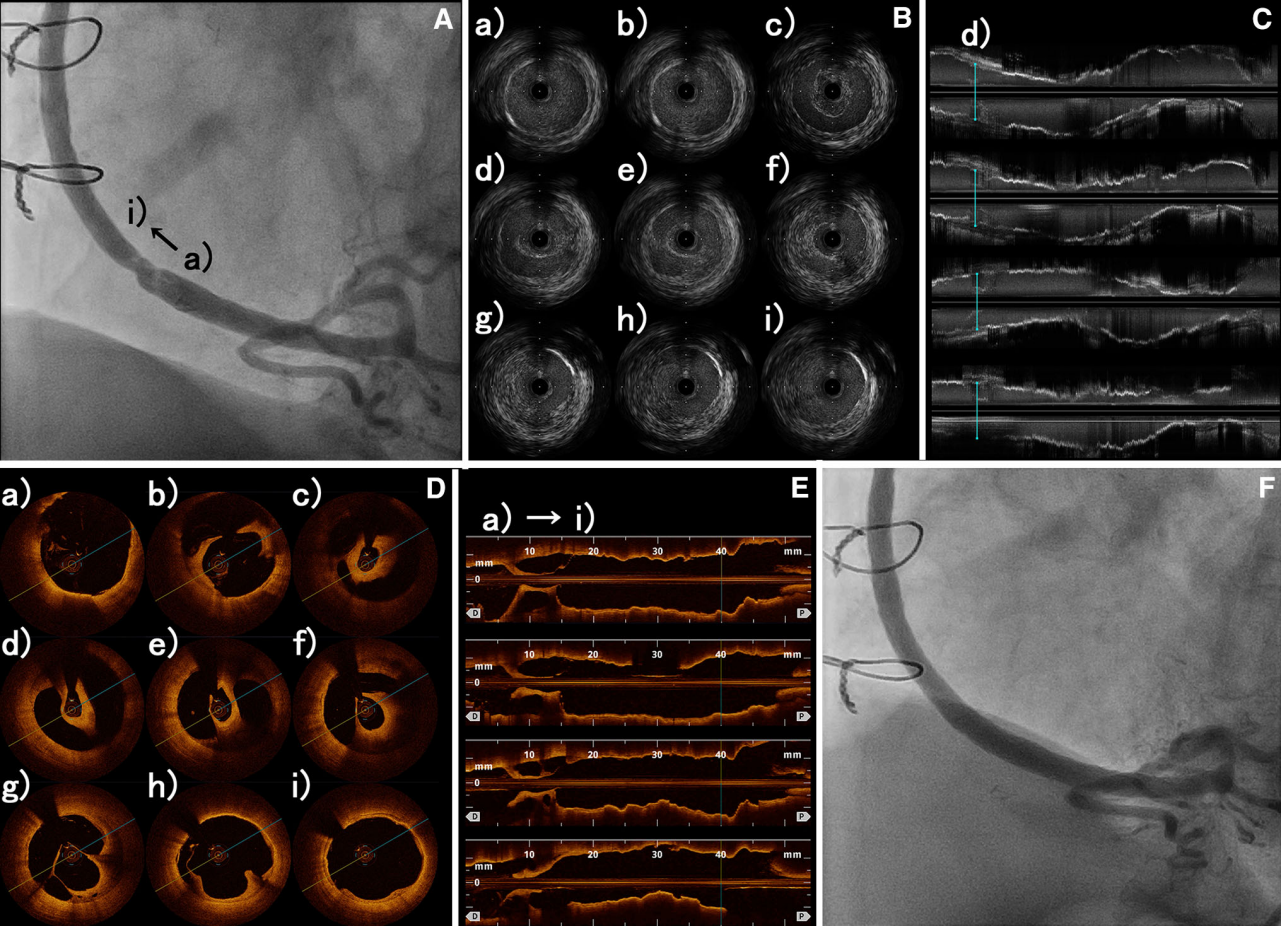
Comparison of intravascular ultrasonography (IVUS) and optical coherence tomography (OCT) images of the culprit lesion. **A** The saphenous vein graft (SVG) before percutaneous coronary intervention (PCI). Distal side of the culprit lesion was **a**, and proximal side was **i**. **B**, **C** IVUS, precise observation, and examination of the lesion in this case were insufficient. **D** Images visualized by short-axis OCT view. **E** Image visualized by long-axis OCT view had the appearance of a venous valve located in opposition to the blood flow. **F** The SVG after PCI

**Fig. 4:**
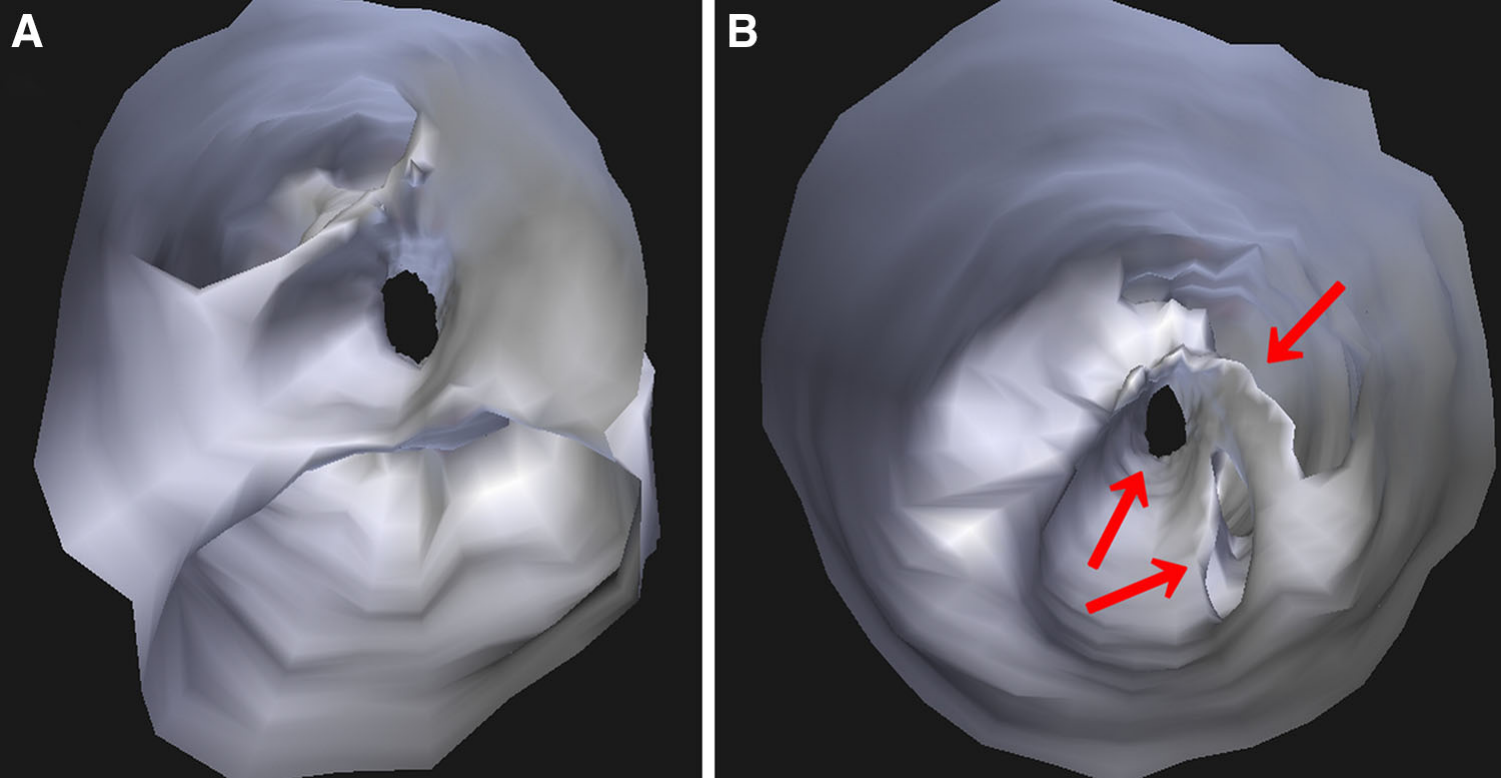
Three-dimensional reconstruction optical coherence tomography (3D-OCT) images of the culprit lesion. The culprit lesion visualized by 3D-OCT images seen from **a** the right coronary artery side and **b** the aorta side. There are at least three pass lines distally from the proximal site (*right arrow*)

**Fig. 5:**
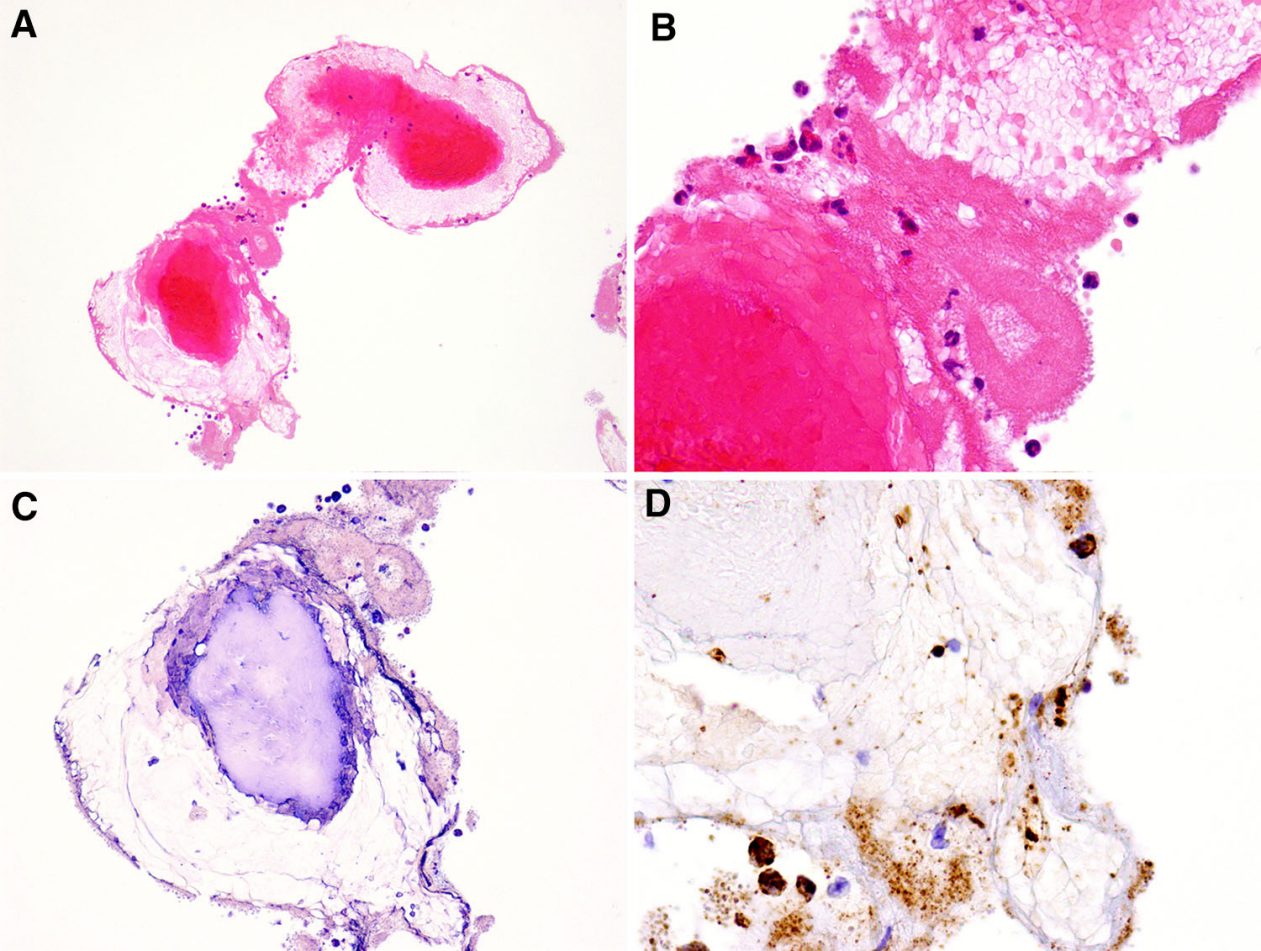
Pathologic images of the thrombus trapped in the filter device. **a** Thrombus consisted of red corpuscles and blood platelets (hematoxylin–eosin (H&E), ×10). **b** There were also a few neutrophils and eosinophils (H&E, ×40). **c** Phosphotungstic acid hematoxylin-positive fibrin (×20). **d** CD68-positive macrophages (×40)

In conclusion, OCT is a very useful imaging modality for clarification of the nature of angiographically ambiguous lesions, as it sheds light on the potential mechanisms and physiology of coronary artery and graft disease progression.
